# Visual Behavior, Pupil Dilation, and Ability to Identify Emotions From Facial Expressions After Stroke

**DOI:** 10.3389/fneur.2019.01415

**Published:** 2020-02-06

**Authors:** Anny Maza, Belén Moliner, Joan Ferri, Roberto Llorens

**Affiliations:** ^1^Neurorehabilitation and Brain Research Group, Instituto de Investigación e Innovación en Bioingeniería, Universitat Politècnica de València, Valencia, Spain; ^2^NEURORHB, Servicio de Neurorrehabilitación de Hospitales Vithas, Valencia, Spain

**Keywords:** social cognition, theory of mind, facial expressions, emotion, visual behavior, gaze, pupil dilation, stroke

## Abstract

Social cognition is the innate human ability to interpret the emotional state of others from contextual verbal and non-verbal information, and to self-regulate accordingly. Facial expressions are one of the most relevant sources of non-verbal communication, and their interpretation has been extensively investigated in the literature, using both behavioral and physiological measures, such as those derived from visual activity and visual responses. The decoding of facial expressions of emotion is performed by conscious and unconscious cognitive processes that involve a complex brain network that can be damaged after cerebrovascular accidents. A diminished ability to identify facial expressions of emotion has been reported after stroke, which has traditionally been attributed to impaired emotional processing. While this can be true, an alteration in visual behavior after brain injury could also negatively contribute to this ability. This study investigated the accuracy, distribution of responses, visual behavior, and pupil dilation of individuals with stroke while identifying emotional facial expressions. Our results corroborated impaired performance after stroke and exhibited decreased attention to the eyes, evidenced by a diminished time and number of fixations made in this area in comparison to healthy subjects and comparable pupil dilation. The differences in visual behavior reached statistical significance in some emotions when comparing individuals with stroke with impaired performance with healthy subjects, but not when individuals post-stroke with comparable performance were considered. The performance dependence of visual behavior, although not determinant, might indicate that altered visual behavior could be a negatively contributing factor for emotion recognition from facial expressions.

## Introduction

Social cognition is the innate human ability to interpret others' feelings and emotions and to regulate one's own behavior accordingly (1). This ability involves a combination of conscious and unconscious processes that facilitate social behavior and has supported human evolution from our ape-like ancestors to our current status as humans (2). Both verbal and non-verbal forms of communication during social interaction are intertwined and reinforced to enable an interpretation of the social context. Body posture (3) and movements (4) and, especially, facial expressions (5, 6) are common sources of non-verbal information that allow us to identify, and discriminate between, to a certain degree, the emotional states of others. Specifically, the ability to recognize emotional expressions on faces has been repeatedly investigated in the literature, evidencing certain universal patterns across cultures (7), ages (8), and sex (9). The recording and analysis of eye movements and gaze patterns through eye-tracking technology have provided cognitive neuroscientists with insights into both the cognitive and physiological processing of visual information (10), which is especially interesting in terms of investigating the ability to recognize facial expression of emotion. Thus, eye-tracking studies have consistently shown that the eyes, mouth, and nose are the most thoroughly explored facial structures involved in scrutinizing emotional expressions (11–13). Moreover, the visual exploration of these areas has been shown to be dependent on the expressed emotion (13, 14), its intensity (11), the visual perspective of the face (15), or the resolution (12) and size of the visual stimuli (16). Apart from visual behavior, eye-tracking technology also allows the temporal variation of pupil size to be registered. Pupil dilation is controlled by both the sympathetic and parasympathetic nervous system (17) in response not only to light changes (18), but also to cognitive processes that involve alertness (19), memory (20), language (21), decision making (22), and emotional processing (18, 23–27). In the latter category, variations in pupil size have been described during the visualization of pictures with emotional attributes in comparison to neutral pictures (24, 26), and similar results have been reported with auditory stimulation (23). Importantly, pupil dilation has been related to an increase in sympathetic activity during emotional processing (24).

The acquisition, processing, and recognition of emotional information from faces involve a complex network of peripheral and central systems. In addition to the visual cortex and cortical association areas, which are commonly involved in the processing of visual information (17, 28), other brain regions, such as the fusiform face area in the ventral temporal lobes, are recruited when a human face is within sight (29). Other structures, such as the inferior occipital gyrus, the superior temporal sulcus (30), and the amygdala (31), are likewise engaged in the decoding of emotional information. The distributed nature of this brain circuitry is particularly vulnerable to both focal and diffuse injuries, such as those derived from cerebrovascular and traumatic accidents, which is supported by the high incidence of impairment in the ability to discriminate among emotions after an injury to the brain (32–44). The great majority of studies on facial emotion recognition have focused on individuals with traumatic brain injury (35–42), and have evidenced an apparent increased difficulty to recognize negative expressions, such as anger, disgust, sadness, or fear (35). Fewer, but still a substantial number of, studies have investigated this ability after stroke (34, 43–45), showing worsened performance in those subjects with lesions in the right hemisphere (34). Concurrent with impaired performance, altered visual exploration behavior has also been reported after brain injuries of different severity (46, 47).

The clinical relevance of difficulties in identifying facial expressions relies on its association with different neurobehavioral symptoms that range from changes in personality (32–42) to impaired self-awareness (48), which can negatively impact social integration (49, 50). These sequelae and other neurobehavioral changes after an acquired brain injury may complicate the quality of life of both the patients and their caregivers (51).

The diminished ability to identify facial expression of emotions after brain lesions has traditionally been explained by an impaired emotional processing (33); however, alterations in visual exploration could bias the integration of visual information and, consequently, have an additional negative effect on the performance of emotional tasks. While this hypothesis has been investigated in other pathologies with associated social cognition deficits, such as schizophrenia (52) and autism spectrum disorders (53), its plausibility after cerebrovascular injury is still unknown.

In light of the existing evidence, we hypothesized that individuals with stroke would perform poorly in comparison to healthy subjects at identifying emotions from facial expressions, and that this effect would also be revealed when considering individual emotions separately. We additionally hypothesized that impaired performance after stroke could be partially explained by an altered visual exploration of the face, and evidenced by an altered variation in pupil dilation. Consequently, the objectives of this study were to investigate the accuracy of the performance, the visual behavior, and pupil dilation of a sample of individuals with stroke during the identification of emotional facial expressions.

## Methods

### Participants

A convenience sample of individuals with stroke were recruited from the outpatient unit of the neurorehabilitation service of Vithas Hospital Valencia al Mar (València, Spain), Vithas Hospital Aguas Vivas (Carcaixent, Spain), and the Brain Injury Center of Vithas Vinalopó (Elx, Spain). The inclusion criteria in this group were diagnosis of stroke confirmed by CT and/or MRI, aged over 18 years, with a fairly good cognitive condition, as defined by scores above 23 in the Mini-Mental State Examination (54), and the ability to follow instructions, as defined by scores above 45 in the receptive language index of the Mississippi Aphasia Screening Test (55). Individuals were excluded if they had disabling visual deficits, such as hemianopsia or impaired visual acuity, which would prevent appropriate visual stimulation and interaction. An additional group of healthy subjects, over 18 years of age, with no known cognitive or psychiatric impairments, were enrolled as controls.

A total of 111 individuals, 46 with stroke and 65 healthy controls, participated in the study. The group of individuals with stroke—either ischemic (*n* = 18) or hemorrhagic (*n* = 28)—consisted of 23 women and 23 men with a median time since injury of 428.0 (222–678) days and a median age of 53.5 (44–58) years. The control group consisted of 35 women and 30 men, with a median age of 48 (30–79) years. No significant differences were found in any demographic variable between these groups (Table 1).

**Table 1:** Characteristics of healthy subjects and individuals with stroke.

	**Healthy subjects**	**Individuals with stroke**	**Significance**
Sex (*n*, %)			*p* = 0.836
Women	35 (53.8%)	23 (50.0%)	
Men	30 (46.2%)	23 (50.0%)	
Age (years)	48.0 (36–62)	53.5 (44–58)	*p* = 0.382
Etiology (*n*, %)	–		–
Ischemic		18 (39.1%)^a^	
TACI		9 (47.4%)^b^	
PACI		5 (26.3%)^b^	
LACI		5 (26.3%)^b^	
Hemorrhagic		28 (60.9%)^a^	
Localization of the injury (*n*, %)	–		–
Right anterior circulation		20 (43.5%)	
Left anterior circulation		17 (37.0%)	
Posterior circulation		9 (19.5%)	
Time since injury (days)	–	428.0 (222–678)	–
Visual perception and cognition	–		–
Letter cancelation test (*n*)		10.0 (10–10)	
Wechsler Memory Scale IV			
Visual reproduction		8.0 (7–9)	
Rey–Osterrieth complex figure copy		32.0 (30–34)	
Color trail test			
Part A (s)		52.0 (38–68)	
Part B (s)		110.0 (80.5–138)	
Wechsler Adult Intelligence Scale IV			
Symbol search		19.50 (14.25–26)	
Matrix reasoning		17.5 (12–22)	

*Demographic and clinical characteristics of healthy participants and individuals with a stroke. TACI, Total anterior circulation infarcts; PACI, Partial anterior circulation infarcts; LACI, Lacunar circulation infarcts. Age, time since injury, and performance in the neuropsychological tests are expressed in terms of median and interquartile range*.

a *Percentage of all participants with stroke*.

b *Percentage of participants with ischemic stroke*.

All subjects who satisfied the inclusion criteria and accepted the terms of participation in the study provided informed written consent before enrolment. Ethical approval for this study was obtained from the Ethics Committees of the clinical institutions involved.

### Instrumentation

Gaze behavior and pupil dilation was estimated using a Tobii TX300 screen-based eye tracker (Tobii AB, Stockholm, Sweden). This device captures gaze data from the corneal reflection of emitted IR light at 300 Hz. The system includes a 23″ screen, with a resolution of 1,920 × 1,080 pixels, which provides visual stimulation, and an eye-tracking unit, which includes an array of IR illuminators (transmitters) and sensors (receptors). In addition, the eye tracker is controlled by a dedicated computer, which incorporates a secondary screen that allows the trial to be managed and supervise without the visual stimulation being interfered with.

Visual stimuli were designed using Tobii Studio 3.2.1 (Tobii AB, Stockholm, Sweden). These consisted of 28 images extracted from the Karolinska Directed Emotional Faces database (56). The images illustrated four subjects—two men and two women—randomly selected from a list of 70 available people. The images reflected facial expressions of fear, anger, disgust, happiness, sadness, surprise, or an absence of emotion (neutrality). The images were displayed in the center of the screen, covering its entire height, which resulted in a picture size of 21 × 28 cm. The remaining areas of the screen were black. It was reported that the minimum time to explore the entire face is 4 s (57). Taking this into account, the stimuli were designed to be displayed in a randomized order for a 5-s period, 1 s longer than the minimum time period necessary to explore the entire face (57), during which gaze behavior and pupil dilation were recorded. Before each image was shown, a black screen was displayed for 500 ms to provide a subtractive baseline correction (58). After each image was shown, a thumbnail of the picture, along with seven words corresponding to the seven possible emotions, were displayed for a maximum of 30 s.

### Procedure

The experiment took place in a dedicated, quiet room in one of the three clinical facilities, which was free of distractors and had controlled lighting conditions. The same experimenter conducted the study at all three sites. The participants were briefly introduced to the task, and were then asked to sit comfortably in a chair facing toward the eye tracker, with their head at an approximate distance of 65 cm from the screen. The eye tracker was calibrated for each participant. After the calibration process, the accuracy of the calibration was experimentally determined, using the deviation between target points on the screen and superimposed estimated fixation points. If the accuracy proved insufficient, the calibration process was repeated. Once the calibration was successful, the experiment was started. The participants were asked to stare at the faces that appeared on the screen for 5 s and then to identify the emotion that, according to their criteria, best matched each facial expression, and to choose this from the seven words shown on the screen. The participants were asked to name the emotion, and the experimenter noted down each answer and then continued the study. If the participants were not able to answer in 30 s, that picture was considered unanswered and the experiment continued. Consequently, the total duration of the study, without considering the calibration process, varied according to the time each participant needed to identify each emotion.

The participants were also assessed using a battery of neuropsychological tests that evaluated the cognitive abilities that involved their visual perceptive skills. This assessment included the letter cancelation test, the visual reproduction subtest of the Wechsler Memory Scale IV, the Rey–Osterrieth complex figure, the color trail test, and the symbol search and matrix reasoning subtests of the Wechsler Adult Intelligence Scale IV.

### Data Analysis

The accuracy in identifying the emotions from the facial expressions was estimated as a percentage of the correct identifications of each emotion, as in previous works (8, 11, 12, 15, 16). According to this value, two subgroups of the individuals with stroke were determined—those with comparable performance to the healthy individuals (those with an equal or better performance than the median performance of the healthy controls) or those with poorer performance than the healthy subjects (those with poorer performance than the median performance of the healthy controls).

Gaze behavior was defined in terms of the number of fixations—also known as fixation count—and the total time spent—also known as total fixation duration—on the eyes, nose, and mouth, which, as mentioned above, have been identified as being the most representative areas involved in a visual scan of the face (11, 12, 14, 15, 59). These areas were manually defined for each visual stimulus image, in accordance with previous studies (11, 12, 15, 59). The averaged pupil diameter variation was also extracted, as in previous studies (18, 60, 61). The results of all the eye-tracking measures represent the averaged behavior of both eyes. Finally, performance at identifying emotions from facial expressions was defined as the percentage of correct identifications.

Prior to the computation of pupil dilation, the pupil data were pre-processed, as follows. First, those images or baselines that presented a ratio of missing data >50% in either eye were discarded (23, 60, 62, 63). Second, the first 2 s of the stimuli were also discarded to remove the initial pupil contraction (60). Third, the non-physiological variations in pupil size, identified as those changes occurring at a faster rate than 5 mm/s, were removed. Fourth, the remaining time windows of missing data were linearly interpolated (23, 62, 63). Fifth, the time series were low-pass filtered at 8.3 Hz to reveal the low-frequency trend (23, 62). Finally, variations in pupil size were obtained through subtractive baseline correction, in which pupil size is converted to an absolute difference from baseline pupil size to that during the stimuli (corrected pupil size = pupil size – baseline) (58).

Differences between the groups of participants, in terms of demographic and clinical variables, visual behavior, and performance, were investigated using independent-sample Mann–Whitney *U* tests, except for sex distribution, etiology, and laterality of injury, which were investigated using chi-squared tests. The level of alpha was set to 0.05 for all analyses.

Data regarding fixation duration, fixation count, and pupil dilation were extracted using Tobii Studio 3.2.1. Signal processing was performed using MATLAB 2018b (MathWorks Inc., MA, USA). The statistical analyses were performed using SPSS for Windows v.22 (IBM, Armonk, NY, USA).

## Results

### Accuracy

**Figure 1:**
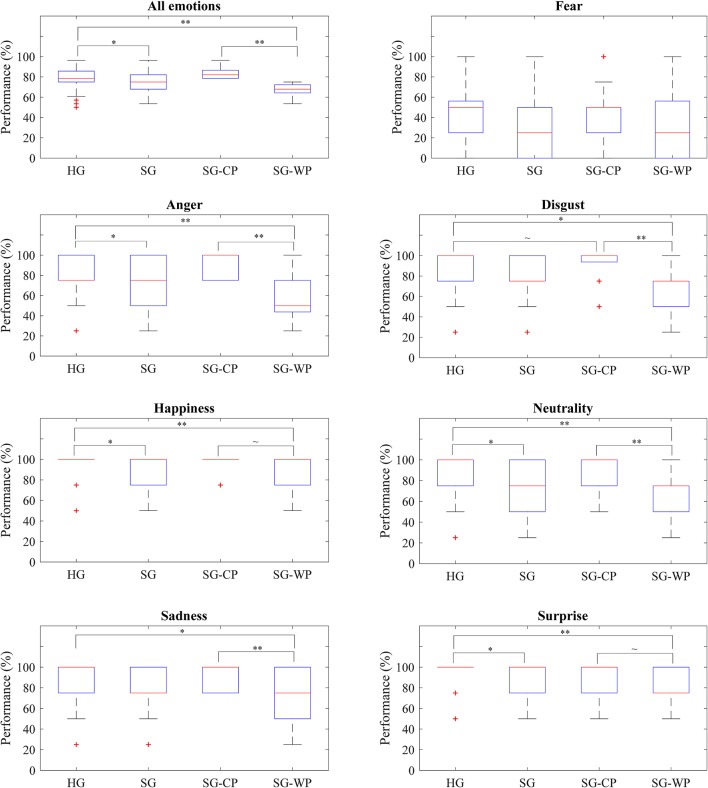
Accuracy of each group of participants by emotion. Percentage of correct answers obtained by all groups of participants in all emotions and in each emotion, separately. HG, Healthy participants; SG, Participants with stroke; SG-CP, Participants with stroke with comparable performance to healthy participants; SG-WP, Participants with stroke with worse performance than healthy participants. **p < 0.01, *p < 0.05, ~*p* < 0.1.

The accuracy in identifying facial expressions of emotion showed significant differences between the individuals with stroke and the healthy subjects, with the former showing decreased accuracy (*p* = 0.012) (Figure 1). The detrimental effect of a cerebrovascular accident incident was consistent for all emotions, but was particularly severe for anger (*p* = 0.030), happiness (*p* = 0.034), neutrality (*p* = 0.016), and surprise (*p* = 0.016), where the differences reached statistical significance.

A more in-depth analysis indicated that 21 participants with stroke showed accuracy comparable to the healthy subjects, while the remaining 25 participants had a relatively poor performance (Table 2). Differences in the overall accuracy of this latter group and the healthy controls reached statistical significance (*p* < 0.001); however, no differences in cognitive ability that involved visual perceptive skills were detected, except in part B of the color trail test. When analyzing performance by emotion, the individuals with stroke and poor performance showed significantly decreased accuracy in comparison to the healthy subjects at identifying anger (*p* < 0.001), happiness (*p* = 0.006), neutrality (*p* < 0.001), sadness (*p* = 0.048), and surprise (*p* = 0.002) (Figure 1).

**Table 2:** Characteristics of individuals with stroke grouped according to their performance.

	**Individuals post-stroke with comparable performance**	**Individuals post-stroke with worse performance**	**Significance**		
			**HG vs. SG-CP**	**HG vs. SG-WP**	**SG-CP vs. SG-WP**
Sex (*n*, %)			*p* = 0.520	*p* = 0.242	*p* = 0.236
Women	13 (61.9%)	10 (40.0%)			
Men	8 (38.1%)	15 (60.0%)			
Age (years)	48.0 (37–58)	55 (46–60)	*p* = 0.706	*p* = 0.098	*p* = 0.114
Etiology (*n*, %)			–	–	*p* = 0.864
Ischemic	9 (42.9%)^a^	9 (36.0%)^a^			
TACI	3 (33.3%)^b^	5 (55.6%)^b^			
PACI	2 (22.2%)^b^	3 (33.3%)^b^			
LACI	4 (44.4%)^b^	1 (11.1%)^b^			
Hemorrhagic	12 (57.1%)^a^	16 (64.0%)^a^			
Localization of the injury (*n*, %)			–	–	*p* = 0.410
Right anterior circulation	5 (23.8%)	15 (60.0%)			
Left anterior circulation	9 (42.9%)	8 (32.0%)			
Posterior	7 (33.3%)	2 (8.0%)			
Time since injury (days)	421.0 (230–641)	431.0 (214–1054)	–	–	*p* = 0.700
Visual perception and cognition					
Letter cancelation test (*n*)	10.0 (10–10)	10.0 (10–10)	–	–	*p* = 0.801
Wechsler Memory Scale IV					
Visual reproduction	8.0 (8–9)	8.0 (7–10)	–	–	*p* = 0.851
Rey–Osterrieth complex figure copy	33.0 (31–34)	31.0 (27–34)	–	–	*p* = 0.134
Color trail test					
Part A (s)	43.5 (35–59)	57.0 (38–75)	–	–	*p* = 0.141
Part B (s)	95.5 (78–121)	125 (92–165)	–	–	*p* = 0.034
Wechsler Adult Intelligence Scale IV					
Symbol search	23.5 (14–32)	18.0 (14–26)	–	–	*p* = 0.281
Matrix reasoning	16.0 (11–22)	18.0 (12–22)	–	–	*p* = 0.972

*Demographic and clinical characteristics of healthy participants and individuals with a stroke. HG, healthy subjects. SG-CP, Individuals with stroke with comparable performance to healthy subjects. SG-WP, Individuals with stroke with worse performance than healthy subjects; TACI, Total anterior circulation infarcts; PACI, Partial anterior circulation infarcts; LACI, Lacunar circulation infarcts. Age, time since injury, and performance in the neuropsychological tests are expressed in terms of median and interquartile range*.

a *Percentage of all participants with stroke*.

b *Percentage of participants with ischemic stroke*.

### Visual Behavior

**Figure 2:**
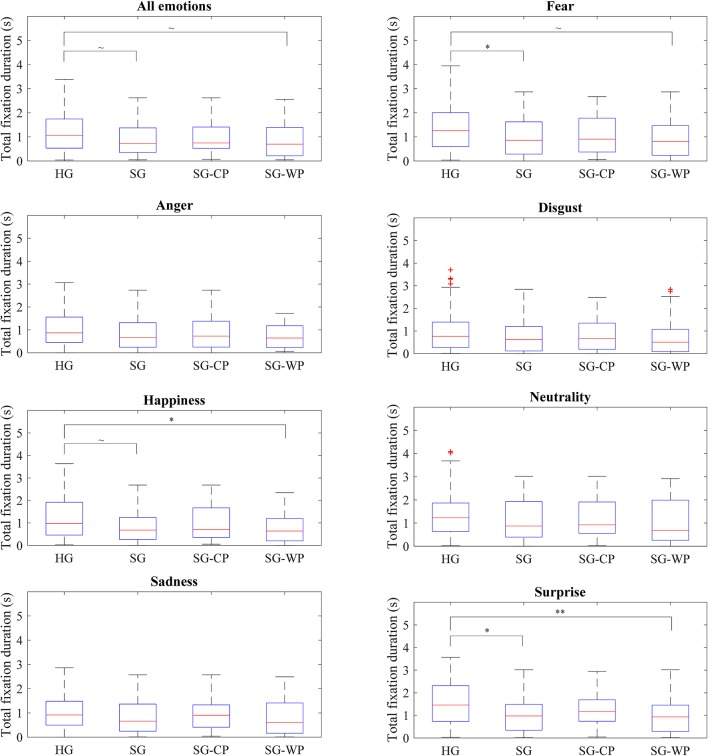
Total fixation duration on the eyes of each group of participants by emotion. Total fixation duration on the eyes showed by all groups of participants in all emotions and in each emotion, separately. HG, Healthy participants; SG, Participants with stroke; SG-CP, Participants with stroke with comparable performance to healthy participants; SG-WP, Participants with stroke with worse performance than healthy participants. **p < 0.01, *p < 0.05, ~*p* < 0.1.

**Figure 3:**
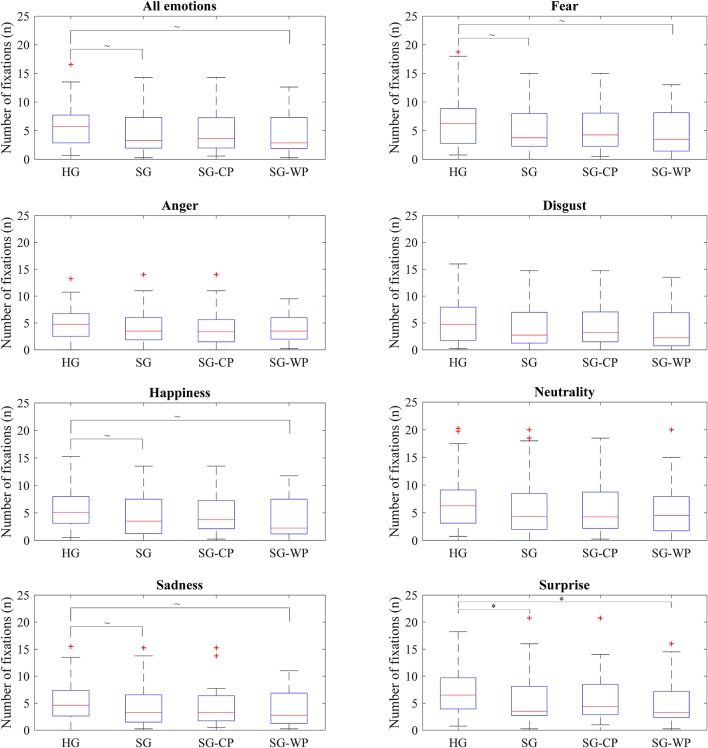
Number of fixations on the eyes of each group of participants by emotion. Number of fixations on the eyes showed by all groups of participants in all emotions and in each emotion, separately. HG, Healthy group; SG, Stroke group; SG-CP, Stroke group with comparable performance; SG-WP, Stroke group with worse performance. *p < 0.05, ~*p* < 0.1.

No significant differences emerged when comparing the visual behavior of the individuals with stroke as a whole and with the healthy subjects; however, the individuals with stroke showed a tendency to spend less time (*p* = 0.073) and perform fewer fixations (*p* = 0.056) on the eyes in comparison to the healthy subjects. While the healthy subjects focused their attention on the eyes, nose, and mouth, in that order, the individuals with stroke mostly focused on the nose rather than the eyes. These differences were consistent for all emotions, and were statistically significant for fear (*p* = 0.039) and surprise (*p* = 0.019) (Figures 2, 3).

No differences in visual behavior were detected between the healthy controls and the participants post-stroke with comparable performance, either when considering all emotions or when analyzing each emotion separately (Figures 2, 3). In contrast, when compared to the healthy controls, individuals post-stroke with poorer performance showed a tendency toward significance in time spent on the eyes (*p* = 0.059) and fixations made on the eyes (*p* = 0.076), both variables having lower values than those of the healthy group. The separate analysis of each emotion showed significant differences between these groups in terms of time spent on the eyes for happiness (*p* = 0.040) and surprise (*p* = 0.008), and in the number of fixations for surprise (*p* = 0.021). Tendencies toward significance appeared in the time spent on the eyes for fear (*p* = 0.053) and in the number of fixations for fear (*p* = 0.053), happiness (*p* = 0.055), and sadness (*p* = 0.092) (Figures 2, 3).

No differences were found between the visual behavior of any group for the mouth or nose.

### Pupil Dilation

**Figure 4:**
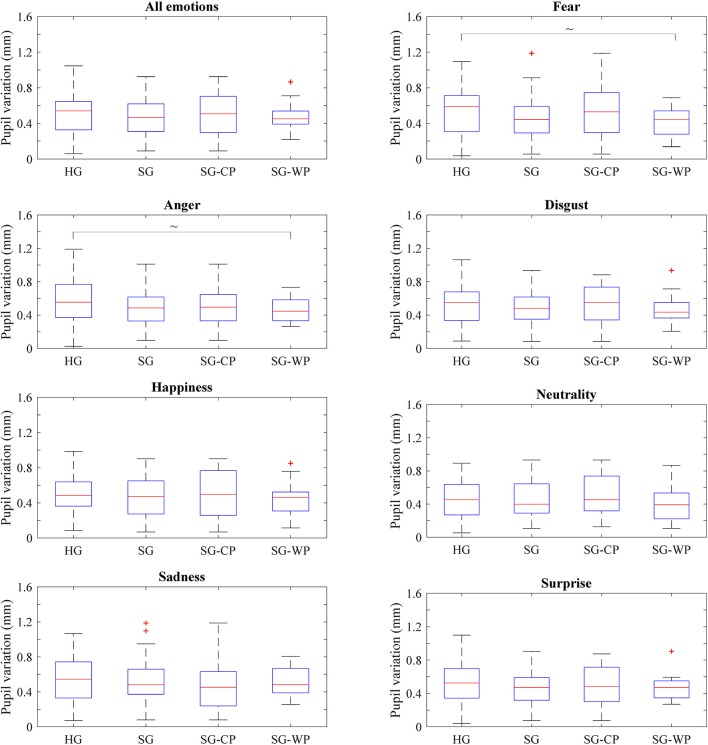
Pupil dilation variation of each group of participants by emotion. Variation of the pupil size showed by all groups of participants in all emotions and in each emotion, separately. HG, Healthy group; SG, Stroke group; SG-CP, Stroke group with comparable performance; SG-WP, Stroke group with worse performance. ~*p* < 0.1.

No significant differences were found in the variation in pupil dilation between the healthy subjects and individuals with stroke, or in general, or by emotion (Figure 4).

No significant differences emerged between the healthy subjects and the individuals with stroke with similar or poorer performance (Figure 4). However, individuals with stroke with poorer performance than the healthy subjects showed a tendency toward signification in fear (*p* = 0.059) and anger (*p* = 0.098) (Figure 4).

## Discussion

This study investigated the accuracy of responses to visual stimuli, the visual behavior, and pupil dilation in individuals with stroke while identifying emotional facial expressions in comparison to healthy subjects. The individuals with stroke showed a significantly relatively poor overall performance in comparison to the healthy subjects, which was also evident when analyzing each emotion separately. Although the different performances between the groups did not correspond to significantly different visual behaviors or pupillary activity, the individuals with stroke seemed to direct less attention toward the eyes and exhibited diminished pupil response. Importantly, when considering those individuals with stroke with impaired performance, these differences were significant for specific emotions. In contrast, the post-stroke individuals with comparable performance to the control group did not show any differences in their visual behavior or pupillary response from those of the healthy subjects. No relevant differences were found between the participants post-stroke with different performance in terms of any demographic or clinical variable, which supports the idea that an impaired ability to identify emotional facial expressions could be partially caused by altered visual behavior.

The ability of the healthy subjects in our study to identify facial expressions of emotion is similar to that reported in previous works, evidencing the greatest accuracy in identifying happiness and surprise, with opposite results in identifying fear (12, 15, 16). Their accuracy was, however, slightly inferior in all emotions in comparison to other reports (12, 15, 16). This effect was especially evident for fear, which had the lowest accuracy values. This might be explained by the fact that the healthy participants in our study, whose ages matched those of the individuals who had had brain injury, were significantly older than the participants in other studies, who were mostly recruited from the student bodies of universities and were, therefore, mostly in their 20s (12, 15, 16). As reported in previous studies, poorer performance at identifying emotional expressions is expected in older age (64, 65). In addition, although the images used in our study were extracted from the same database used in other studies (12, 15, 16), and the images were randomly selected, the emotions shown in the images may have been more difficult to recognize than in the images used in other studies. The visual behavior of the healthy participants was also consistent with existing reports, showing that eyes, noses, and mouths are the most relevant facial structures used in identifying facial expressions (12–15, 59). The hierarchical distribution of attention to the eyes, followed by the nose and the mouth, is also supported in most of the existing literature (11, 15, 16). In this study, the eyes were especially relevant when identifying surprise and fear, but seemed to draw less attention for disgust, which is consistent with a previous study (15). Nonetheless, it is important to highlight that there is no fixed or common pattern of visual behavior while identifying different emotions, as equally evidenced by our study and in previous reports (12, 15, 16). Additionally, our results must be taken into account considering that assessing accuracy by a simple count of the correct identifications without regard for false alarms or bias in the use of response categories, although it is the most common approach to analyze this behavior (8, 11, 12, 15, 16), could be misleading (66).

The variation in pupil dilation in our study was greater than that reported in previous studies (24, 60, 67). This dissimilarity may have derived from the use of different images, which, despite having been normalized, might have promoted different levels of arousal, consequently modulating the pupil response in a different way. Our results are, however, supported by a previous study, which reported the lowest variation in pupil dilation for expressions of happiness and neutrality, and the highest variation for fear (60). Despite this, it should be taken into consideration that variations in pupil dilation are triggered by different mechanisms, from simple autonomous processes, such as pupillary light reflex (18, 19), to high executive functioning (22, 27), so a definitive identification of the source of the variation is not possible using this technology. In addition, although the methodology of our study has been repeatedly used in previous investigations (25, 57, 60), it is important to consider that the use of a black screen as a baseline may have negatively contributed to the identification of the source of the pupil variation.

Individuals with stroke showed impaired performance at identifying facial expressions of emotion, in line with previous studies (34, 43–45). Interestingly, these studies grouped the emotions by their attributes, reporting that individuals post-stroke exhibited better performance at identifying positively attributed emotions over negatively attributed emotions (43–45). This effect is also supported by our results, which showed the greatest accuracy for happiness and surprise, and the worst performance for fear. Nevertheless, the differences between the healthy participants and the individuals with stroke were not significant for all emotions, in contrast to what was reported in a previous study (43). The use of different images might explain these dissimilarities. Some emotions in our study might have been particularly difficult to interpret, affecting both groups in a similar way. The decreased attention toward the eyes exhibited by the individuals post-stroke in comparison to the healthy subjects is suggestive of an altered perception of visual information, which could partially explain their impaired ability to identify the facial expression of emotions. This hypothesis is supported by the differences in visual behavior for happiness and surprise, which represented a huge challenge to this group of participants. In contrast, differences in the accuracy of identifying anger and neutrality were not associated with differences in visual behavior when observing these emotions. The inconsistency in these results might reflect the complexity of the perceptual and cognitive processes underlying the decoding of facial expressions (3, 30). Although not statistically significant, individuals post-stroke showed a slightly diminished pupillary response compared to the healthy subjects, which, if endorsed in further studies, might reflect diminished emotional arousal, confirming previous reports (18, 23, 24). It is important to highlight, however, that pupil dilation is also driven by the co-activation of multiple brain areas (19, 68), which might be affected by a cerebrovascular accident.

In general, a comparison of the visual behavior and pupillary activity between healthy subjects and individuals with stroke only showed a decreased attention to the eyes, but this did not reach statistical significance. Although these results might support a degree of comparability between both groups, a separate analysis of the individuals with stroke, according to their performance, exposed significant differences. Differences between individuals with stroke with impaired performance and healthy subjects were stronger and significant for happiness, surprise, and fear. Pupil dilation was also lower and showed a tendency to significance for fear and anger. In contrast, participants with comparable performance showed similar visual behaviors and pupillary responses. The differences detected in groups with different performances, but an absence of any other clinical or demographic dissimilarities, suggest that altered visual behavior could be a contributing factor to impaired performance, rather than the neurological condition itself. Altered visual behavior, together with impaired emotional processing, which has been repeatedly reported after stroke (34, 43), could explain the accuracy of these individuals in identifying emotional facial expressions.

## Conclusions

This study corroborated the negative effect of a cerebrovascular accident on the ability to identify facial expressions of emotion, which was also supported by analyzing the emotions separately. Our results showed that individuals with stroke looked for a shorter time and fewer times at the eyes than did healthy subjects, but without significantly differing from the pattern of observation of the healthy subjects. These differences were, however, accentuated when analyzing individuals with stroke according to their performance. While no differences were detected between the healthy subjects and the individuals post-stroke with comparable performance, this latter group showed increased and significant differences in different measures compared to healthy subjects, suggesting that altered visual behavior might be associated with, and be a contributing factor to, difficulties in identifying the facial expression of emotions after stroke. No significant differences were found in pupil dilation between healthy controls and individuals with stroke.

## Data Availability Statement

The datasets generated for this study will not be made publicly available as they contain confidential data.

## Ethics Statement

Ethical approval for the study was granted by the Institutional Review Board of Vithas Hospital Valencia al Mar. All participants provided written informed consent before taking part in the study.

## Author Contributions

RL designed the study. BM, JF, and RL defined the clinical aspects regarding individuals with stroke, and BM assessed their condition. AM conducted the experimental sessions and analyzed the data. All the authors discussed the results of the experiment.

### Conflict of Interest

The authors declare that the research was conducted in the absence of any commercial or financial relationships that could be construed as a potential conflict of interest.
